# Development and Characterization of Silibinin-Loaded Nanoemulsions: A Promising Mucoadhesive Platform for Enhanced Mucosal Drug Delivery

**DOI:** 10.3390/pharmaceutics17020192

**Published:** 2025-02-04

**Authors:** Ana Paula Santos Tartari, Joslaine Jacumazo, Ariane Krause Padilha Lorenzett, Rilton Alves de Freitas, Rubiana Mara Mainardes

**Affiliations:** 1 Laboratory of Nanostructured Formulations, Universidade Estadual do Centro-Oeste-UNICENTRO, Alameda Élio Antônio Dalla Vecchia, 838, Guarapuava 85040-167, PR, Brazil; ap.tartari@hotmail.com (A.P.S.T.); arianekrausepad@gmail.com (A.K.P.L.); 2 BioPol, Chemistry Department, Universidade Federal do Paraná (UFPR), R. Coronel F. H. dos Santos, 210, Curitiba 81531-980, PR, Brazil; joslainejacumazo@ufpr.br (J.J.); rilton@ufpr.br (R.A.d.F.); 3 Pharmacy Department, Universidade Estadual do Centro-Oeste-UNICENTRO, Alameda Élio Antônio Dalla Vecchia, 838, Guarapuava 85040-167, PR, Brazil

**Keywords:** silybin, nanoemulsion, QMC-D analysis, mucin

## Abstract

**Background:** Silibinin (SLB), a flavonoid derived from milk thistle, exhibits promising therapeutic properties but faces significant clinical limitations due to poor solubility and bioavailability. **Objectives:** This study focuses on the development and characterization of SLB-loaded nanoemulsions designed for mucosal delivery. **Methods:** Nanoemulsions were prepared using the spontaneous emulsification method, guided by pseudoternary phase diagrams to determine selected component ratios. Comprehensive characterization included particle size, polydispersity index (PDI), zeta potential, encapsulation efficiency, rheological properties, and surface tension. Mucoadhesive properties were evaluated using quartz crystal microbalance with dissipation (QCM-D) to quantify interactions with mucin layers. **Results:** The combination of Capryol 90, Tween 80, and Transcutol in selected proportions yielded nanoemulsions with excellent stability and solubilization capacity, enhancing the solubility of silibinin by 625 times compared to its intrinsic solubility in water. The ternary phase diagram indicated that achieving nanoemulsions with particle sizes between 100 and 300 nm required higher concentrations of surfactants (60%), relative to oil (20%) and water (20%), with formulations predominantly composed of Smix (surfactant and cosurfactant mixture in a 1:1 ratio). Rheological analysis revealed Newtonian behavior, characterized by constant viscosity across varying shear rates and a linear torque response, ensuring ease of application and mechanical stability. QCM-D analysis confirmed strong mucoadhesive interactions, with significant frequency and dissipation shifts, indicative of prolonged retention and enhanced mucosal drug delivery. Furthermore, contact angle measurements showed a marked reduction in surface tension upon interaction with mucin, with the SLB-loaded nanoemulsion demonstrating superior wettability and strong mucoadhesive potential. **Conclusions:** These findings underscore the suitability of SLB-loaded nanoemulsions as a robust platform for effective mucosal drug delivery, addressing solubility and bioavailability challenges while enabling prolonged retention and controlled therapeutic release.

## 1. Introduction

Silibinin (SLB), a flavonolignan extracted from *Silybum marianum* (milk thistle), has been extensively studied for its pharmacological properties. This bioactive compound, alongside other flavonolignans such as isosilibinin, silicristin, and silidianin, and the flavonoid taxifolin, confers a range of therapeutic effects, including hepatoprotective, antioxidant, anti-inflammatory, antifibrotic, and anticancer activities [1,2]. Despite these promising properties, the clinical application of SLB remains limited due to its poor water solubility (approximately 0.04 mg/mL), extensive first-pass metabolism, and low oral bioavailability, estimated to be less than 1% [3,4]. These pharmacokinetic barriers hinder its therapeutic potential, emphasizing the need for innovative delivery systems.

Nanoemulsions are a versatile platform for the delivery of poorly water-soluble drugs like SLB. Nanoemulsions are isotropic, thermodynamically or kinetically stable colloidal systems with droplet sizes typically ranging from 20 to 200 nm, composed of an oil phase, an aqueous phase, and surfactants [5,6]. Their nanoscale size provides a high surface area, enhancing the solubilization of lipophilic drugs, protecting them from premature degradation, and facilitating their cellular uptake [7,8]. Moreover, the nanoemulsion system can be engineered to provide controlled release and increased stability under physiological conditions, addressing the key pharmacokinetic challenges of SLB [9].

In addition to improving bioavailability, nanoemulsions can be functionalized for specific drug delivery applications. One particularly promising approach is enhancing mucoadhesion, a property that increases the retention time of the formulation at mucosal sites, such as the gastrointestinal or nasal mucosa. Mucoadhesive systems can interact with mucin glycoproteins via hydrogen bonding, electrostatic interactions, or hydrophobic forces, promoting prolonged drug release and enhancing therapeutic efficacy [10,11]. This feature is particularly relevant for SLB, as it enables targeted delivery to mucosal tissues, reducing systemic side effects and improving local bioavailability.

The formulation method is crucial for the performance of nanoemulsions. Among the available techniques, spontaneous emulsification stands out due to its simplicity and effectiveness. This method involves the rapid formation of nanoemulsion droplets when an oil phase, a surfactant, and an aqueous phase are mixed in appropriate proportions, without the need for external mechanical energy such as sonication or high-pressure homogenization [12,13]. This approach is particularly advantageous for the encapsulation of thermolabile compounds like SLB, as it minimizes the risk of degradation during processing.

To maximize the potential of SLB-loaded nanoemulsions, thorough characterization is essential. Key parameters, such as droplet size, polydispersity index, zeta potential, drug encapsulation efficiency, and stability, must be evaluated to ensure selected performance. Additionally, mucoadhesive properties should be assessed to validate the suitability of the formulation for mucosal drug delivery applications. Such comprehensive characterization not only informs the development of effective SLB delivery systems but also lays the groundwork for expanding their clinical applications [11,14].

This study aims to develop and characterize SLB-loaded nanoemulsions prepared using the spontaneous emulsification method, focusing on their physicochemical properties and mucoadhesive potential. The findings could pave the way for novel, efficient delivery systems for SLB, overcoming its pharmacokinetic challenges and broadening its therapeutic applicability.

## 2. Materials and Methods

### 2.1. Materials

The oils Capryol 90, oleic acid, and Labrafil were generously donated by Gattefossé (Saint-Priest, Rhône-Alpes, France). The surfactants and cosurfactants Tween 80, Tween 20, ethylene glycol, Transcutol, and propylene glycol were purchased from Sigma-Aldrich (St. Louis, MO, USA). Silibinin (SLB) was obtained from Sigma-Aldrich (St. Louis, MO, USA). Dimethyl sulfoxide (DMSO) was obtained from Synth (Diadema, São Paulo, Brazil), and acetonitrile was supplied by JT Baker (Phillipsburg, NJ, USA).

### 2.2. Solubility Studies

The solubility of SLB in various oils and surfactants/cosurfactants was evaluated to select appropriate components for the nanoemulsion formulation. Oils tested included Capryol 90, oleic acid, and Labrafil, while surfactants/cosurfactants included Tween 80, Tween 20, ethylene glycol, propylene glycol, and Transcutol. The calibration curve was constructed using SLB standard concentrations ranging from 1 to 50 mg/mL, with a correlation coefficient (R^2^) exceeding 0.999, ensuring accurate quantification within the evaluated solubility range. Excess SLB was added to 2 mL of each oil or surfactant in 5 mL vials, followed by vortex mixing. Samples were incubated at 25 °C on an isothermal shaker for 72 h to reach equilibrium. After centrifugation at 10,000 rpm for 15 min, the supernatant was collected and analyzed for SLB concentration using UV-Vis spectrophotometry at 288 nm [15].

### 2.3. Hydrophilic–Lipophilic Balance (HLB) Study

To determine the ratio of surfactant and cosurfactant, an in situ emulsification method was used, according to Ahmad et al. [16] with modifications. The system consisted of oil (fixed at 10%), Smix (surfactant and cosurfactant in varying ratios from 1:9 to 9:1), and distilled water. The mixtures were titrated with water and visually assessed for transparency and flowability.

### 2.4. Pseudo-Ternary Phase Diagram Construction

Pseudo-ternary phase diagrams were constructed using the aqueous titration method [16]. Surfactant and cosurfactant mixtures (Smix) were prepared in various ratios, and emulsions were formulated by varying the oil, Smix, and aqueous phase ratios. A total of 59 emulsions were formulated. Phase diagrams were constructed to identify the most stable formulations.

### 2.5. Preparation of Nanoemulsions via Spontaneous Emulsification

Nanoemulsions containing SLB were prepared using the spontaneous emulsification method. SLB (10 mg) was dissolved in the selected oil (600 µL) under stirring at 200 rpm. The Smix system was then gradually added under constant agitation. This oil phase was added dropwise into the aqueous phase under continuous stirring at a controlled rate (2 min). Control emulsions (without SLB) were prepared using the same procedure. The ratios of oil, Smix, and water were determined using pseudo-ternary phase diagrams.

### 2.6. Particle Size, Polydispersity Index (PDI), and Zeta Potential (ZP)

The average particle size and PDI of the nanoemulsions were determined using dynamic light scattering (DLS) with the Brookhaven 90 Plus^®^ instrument (Brookhaven Instruments Corporation, New York, NY, USA). Samples were diluted in ultrapure water at a 1:100 ratio and placed in capped cuvettes. Measurements were performed at a scattering angle of 90° and a temperature of 25 °C, using a laser with a 659 nm wavelength. Results were reported as the mean ± standard deviation (SD) of three independent measurements.

ZP was assessed by measuring the electrophoretic mobility of the particles using a Zetasizer (ZS-Malvern^®^, Malvern Panalytical, Cambridge, UK). Samples were diluted in a 1 mmol·L^−1^ KCl aqueous solution (1:100) and analyzed in an electrophoretic cell. A potential of ±150 mV was applied, and results were reported as the mean ± SD from triplicate measurements.

### 2.7. Encapsulation Efficiency Determination

The encapsulation efficiency (EE) of SLB was determined by a direct method using the following equation:

$$EE\;\left(\%\right)=\frac{amount\;of\;encapsulated\;SLB}{amount\;of\;SLB\;added}\times 100$$

To perform the analysis, nanoemulsions were diluted at a 1:8 ratio in dimethyl sulfoxide (DMSO) to disrupt the emulsion structure and release the encapsulated drug. The diluted samples were then subjected to centrifugation at 25,000 rpm for 45 min to isolate the drug content effectively. The supernatant was filtered through 0.45 μm hydrophobic PVDF filters (Millipore^®^, Burlington, MA, USA) and analyzed by ultra-performance liquid chromatography coupled with tandem mass spectrometry (UPLC/MS-MS) (XEVO-TQD, Waters^®^, Milford, MA, USA) equipped with a Z sprayTM electrospray ionization source (Waters^®^, Milford, MA, USA). The mobile phase consisted of acetonitrile and 0.5% acidified water (with formic acid) in isocratic mode, with a flow rate of 1 mL·min^−1^ and a column temperature at 40 °C. To detect SLB, mass spectrometry was employed in negative electrospray ionization mode (ESI-). The mass spectrometry conditions incorporated a capillary voltage of 3.00 kV, a cone voltage of 60 V, an extractor voltage of 3.0 V, a source temperature of 150 °C, and a desolvation temperature of 600 °C. The quantification method relied on multiple reaction monitoring (MRM) mode, with the transitions set at *m*/*z* 481.09 → 151.90 [17].

### 2.8. Stability Evaluation

The stability of SLB-loaded nanoemulsions was assessed through kinetic and storage stability tests. Kinetic stability was evaluated by centrifugation at 5000 rpm for 30 min at 25 °C. Storage stability was assessed at room temperature and refrigeration (4–10 °C) over six months. Changes in droplet size were monitored.

### 2.9. Quantitative Mucoadhesion Analysis Using QCM-D

To assess the mucoadhesive properties of the nanoemulsion, a quartz crystal microbalance with dissipation (QCM-D) was used (Biolin Scientific, Q-Sense E4—Manchester, UK). Analyses were conducted in triplicate using the flow cell mode. Gold-coated QCM-D sensors were cleaned before use or reuse by immersion in a 1:1:5 mixture of hydrogen peroxide (30% *v*/*v*), ammonium hydroxide (25% *v*/*v*), and ultrapurified water for 10 min at 75 °C, followed by rinsing with ultrapurified water, drying with nitrogen, rinsing with ethanol (99%), and drying with nitrogen gas. This cleaning procedure was repeated at least three times. The surface modification procedure followed a standard protocol provided by Q-Sense [18].

The surface modification procedure included the following steps: (1) before mucin layer deposition at 25 mg·L^−1^, the buffer was injected into the flow cell to establish a stable baseline; (2) after signal stabilization, mucin at 25 mg·L^−1^ was introduced onto the crystal until both the frequency (Δf) and dissipation (ΔD) stabilized; (3) the buffer was rinsed for approximately 10 min to remove unbound mucin; (4) after establishing the mucin layer, nanoemulsions (diluted in distilled water 1:100) were introduced into the measurement cell for approximately 60 min; and (5) finally, unbound emulsions were removed by washing with the buffer for 10 min [19].

### 2.10. Rheological Properties

Rheological properties were evaluated using a rotational rheometer (HR10 Discovery, TA Instruments, New Castle, DE, USA) equipped with cone-plate geometry (cone diameter: 40 mm; cone angle: 2°). Oscillatory shear and amplitude sweeps were conducted at stress ranges of 1 × 10^−3^ Pa to 20.0 Pa and temperatures between 10 °C and 50 °C [20].

### 2.11. Surface Tension Determination

Surface tension was measured using pendant drop tensiometry (DataPhysics Instruments GmbH, Filderstadt, Germany). Images of droplets were captured, and profiles were analyzed using the ellipsoid model to determine contact angles and surface tension [21].

## 3. Results and Discussion

### 3.1. SLB Solubility Testing

**Figure 1:**
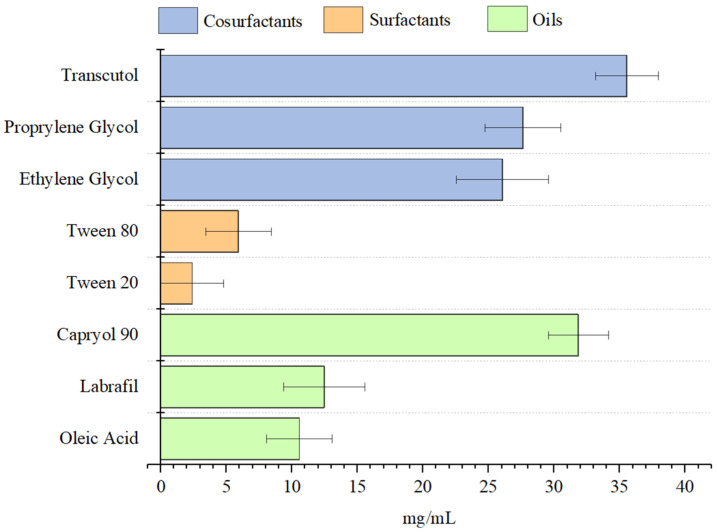
Solubility of SLB in various oils, surfactants, and cosurfactants (mg/mL).

The solubility of SLB in different oils, surfactants, and cosurfactants is illustrated in Figure 1. The results demonstrate significant variability among the tested compounds, with Capryol 90, Transcutol, and propylene glycol exhibiting the highest solubilization capacities, reaching or exceeding 25 mg/mL. This represents an improvement of over 625 times in the solubility of silibinin compared to its intrinsic solubility in water (0.04 mg/mL). These solvents are followed by ethylene glycol and oleic acid, while Labrafil and surfactants like Tween 80 and Tween 20 presented lower solubility values.

#### 3.1.1. Solubility in Cosurfactant

Transcutol exhibited the highest solubilization capacity for SLB, with values exceeding 30 mg/mL, demonstrating significant superiority compared to propylene glycol and ethylene glycol. Transcutol demonstrated a similarly high capacity to dissolve SLB due to its amphiphilic properties, allowing it to solubilize both lipophilic and hydrophilic compounds. Its penetration-enhancing capabilities further support its use in drug delivery systems [22,23]. Conversely, while propylene glycol and ethylene glycol displayed good solubilization capacities, their lower values suggest limitations in molecular interactions with SLB, likely due to structural differences and reduced chemical affinity with the flavonoid molecule.

These findings highlight the critical role of solvent selection in the formulation of SLB-loaded nanoemulsions. Cosurfactants such as Transcutol not only increase the solubility but also improve the stability and dispersibility of bioactive compounds in colloidal systems. This highlights their relevance in the development of effective nanoemulsions to enhance the therapeutic performance of SLB.

#### 3.1.2. Solubility in Surfactant

Among the surfactants tested, Tween 80 showed superior solubilization performance compared to Tween 20, likely due to its higher HLB value, which facilitates the encapsulation of hydrophobic drugs like SLB within micelles [23,24]. Nonionic surfactants, such as Tween 80, are widely used in pharmaceutical formulations for their ability to reduce interfacial tension and form stable micellar systems. Their lack of ionic interactions further enhances their compatibility in various formulation conditions [25].

#### 3.1.3. Solubility in Oil

The superior performance of Capryol 90 can be attributed to its chemical composition as a medium-chain triglyceride derived from caprylic acid. This structure provides a highly lipophilic environment that effectively interacts with hydrophobic molecules such as SLB [26]. Moreover, medium-chain monoglycerides like Capryol 90 are known for their excellent emulsification properties, making them more effective than their long-chain counterparts in forming stable emulsions [27].

On the other hand, Labrafil showed lower solubilization capacity, indicating reduced compatibility with the chemical structure of SLB. Although Labrafil contains mixed glyceride esters, its higher molecular weight may reduce its efficiency in interacting with SLB compared to lighter compounds.

### 3.2. Required HLB Study

The spontaneous emulsification process is influenced by multiple factors, including the surfactant-to-cosurfactant ratio, the composition of the oily phase, and the thermodynamic conditions of the system. One critical parameter for optimizing this process is the HLB, which quantifies the balance between the hydrophilic and lipophilic portions of surfactant molecules. This balance determines the surfactant’s affinity for oil and water, making it fundamental in the formulation of stable oil-in-water (O/W) emulsions [28].

In this study, 17 formulations with varying surfactant-to-cosurfactant ratios were evaluated to identify the selected HLB value for nanoemulsion stability. Table 1 summarizes the results for all tested formulations, including their HLB values, visual appearance, particle size, PDI, and zeta potential, considering different proportions of Smix (Tween 80 and Transcutol). Among these, the 1:1 ratio (HLB = 9.6) exhibited the best performance, producing a translucent emulsion without phase separation. This formulation achieved an average particle size of 343.0 nm, a low polydispersity index (PDI) of 0.351, and a zeta potential of −9.66 mV. These characteristics indicate a uniform droplet size distribution and suggest favorable conditions for long-term stability, as nanoemulsions with narrow size distributions are less prone to destabilizing phenomena such as coalescence, creaming, or sedimentation [11].

The absence of phase separation in the 1:1 formulation can be attributed to the effective interaction between surfactants and dispersed phases, reducing interfacial tension and promoting system stability. Effective emulsifiers, particularly those with HLB values between 8 and 18, are ideal for O/W emulsions as they provide a balance between hydrophilic and lipophilic interactions, ensuring stable emulsions with minimal phase separation tendencies [29].

**Figure 2:**
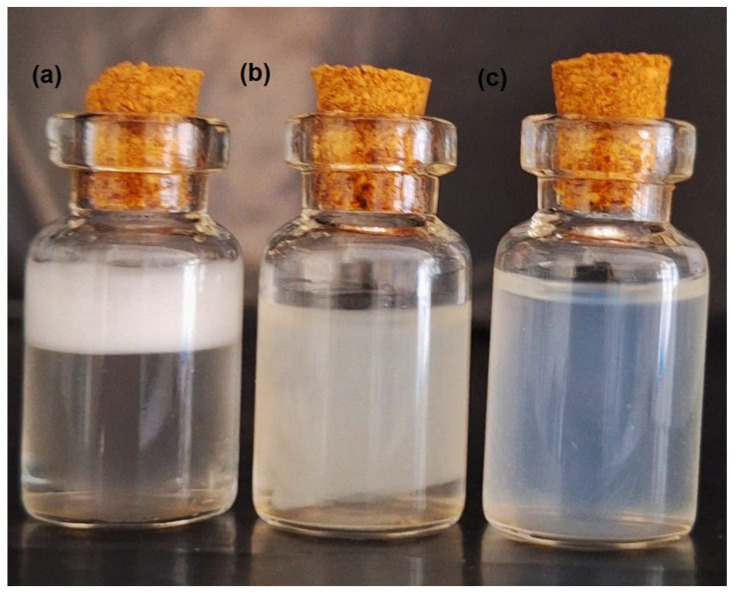
(**a**) Translucent emulsion with phase separation, (**b**) white emulsion with phase separation, (**c**) translucent emulsion without phase separation.

In contrast, formulations with other HLB values showed varying degrees of instability (Figure 2). Ratios with lower HLB values (e.g., 1:6 or 1:8) and higher HLB values (e.g., 3:1 or 7:1) resulted in emulsions with visible phase separation, larger particle sizes, and higher PDIs, indicating less stable systems. These observations highlight the importance of tailoring the HLB value to achieve the selected surfactant balance and emulsion performance.

### 3.3. Construction and Analysis of the Ternary Phase Diagram

**Figure 3:**
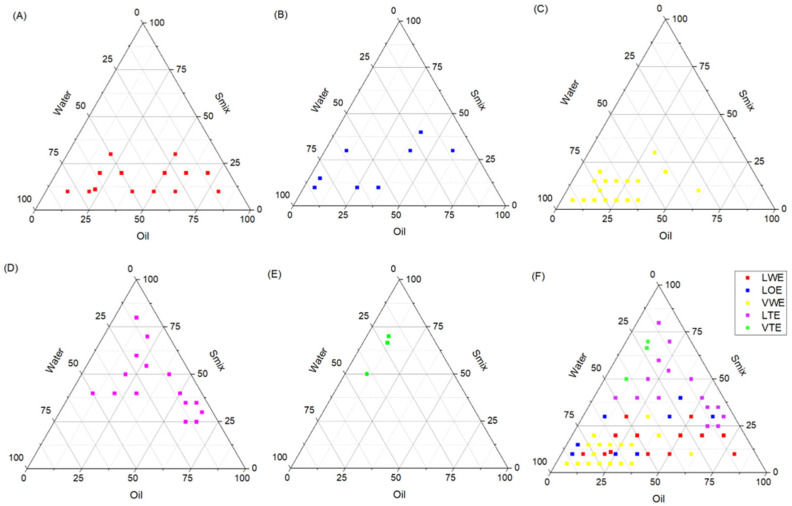
Ternary phase diagram of 59 emulsion formulations categorized by visual and physical properties based on proportions of water, oil (Capryol 90), and Smix. Each point on the diagram corresponds to a specific formulation, with different colors indicating various properties of the emulsions: (**A**) LWE (red) for liquid and white emulsions, (**B**) LOE (blue) for liquid and opaque emulsions, (**C**) VWE (yellow) for viscous and white emulsions, (**D**) LTE (purple) for liquid and translucent emulsions, (**E**) VTE (green) for viscous and translucent emulsions, and (**F**) represents all points.

Figure 3 illustrates the ternary phase diagram for the 59 tested formulations, mapping the proportions of water, oil (Capryol 90), and the surfactant/cosurfactant mixture (Smix). Each data point represents a specific formulation, categorized into five groups based on their visual and physical properties: liquid white emulsions (LWEs, red), liquid opaque emulsions (LOEs, blue), viscous white emulsions (VWEs, yellow), liquid translucent emulsions (LTEs, purple), and viscous translucent emulsions (VTEs, green).

The analysis of the diagram reveals that formulations classified as LWE are primarily located in regions with higher water content and lower Smix concentrations. These emulsions exhibit a white appearance and liquid consistency, suggesting limited stability due to insufficient surfactant–cosurfactant interactions to adequately reduce interfacial tension and stabilize oil droplets. As a result, phase separation is more likely to occur in these formulations.

In contrast, LOE formulations are situated in areas with intermediate Smix concentrations and moderate water content. The opaque appearance of these emulsions indicates partial stabilization; however, their long-term stability may remain inadequate due to uneven droplet size distribution or suboptimal Smix proportions.

Formulations categorized as VWE are found in regions dominated by high oil content and low Smix proportions. These emulsions are characterized by increased viscosity and a white appearance, reflecting challenges in achieving proper emulsification. The lack of translucency in this group highlights the presence of non-uniform droplet sizes, which compromise stability and hinder their application as nanoemulsions.

On the other hand, LTE formulations, which are in regions with a balanced composition of water, oil, and Smix, demonstrate superior properties. These emulsions are translucent and exhibit a liquid consistency, indicative of successful nanoemulsion formation. The uniform droplet size distribution and high stability observed in this category make them ideal candidates for drug delivery applications, where both physicochemical stability and bioavailability are critical.

Finally, VTE formulations are observed in regions with relatively high Smix content and moderate proportions of oil and water. These emulsions are translucent with increased viscosity, suggesting enhanced stability due to the prevention of droplet coalescence. The favorable properties of this group indicate their potential for controlled-release systems and prolonged stability.

Overall, the data presented in the ternary phase diagram highlight the crucial role of balancing the proportions of water, oil, and Smix to achieve stable and effective emulsions. The regions associated with LTE and VTE formulations demonstrate that adequate Smix content is essential to reduce interfacial tension, maintain small and uniform droplet sizes, and prevent phase separation. Conversely, the regions yielding LWE and VWE emulsions emphasize the limitations of compositions with insufficient surfactant/cosurfactant or excessive oil content, leading to poor emulsification and instability.

The results further underscore the importance of systematically optimizing the composition of nanoemulsions to ensure their suitability for pharmaceutical applications. By providing a visual representation of the formulation space, the ternary phase diagram serves as a valuable tool for identifying the ideal ranges of components required to produce high-quality nanoemulsions with desired characteristics.

**Figure 4:**
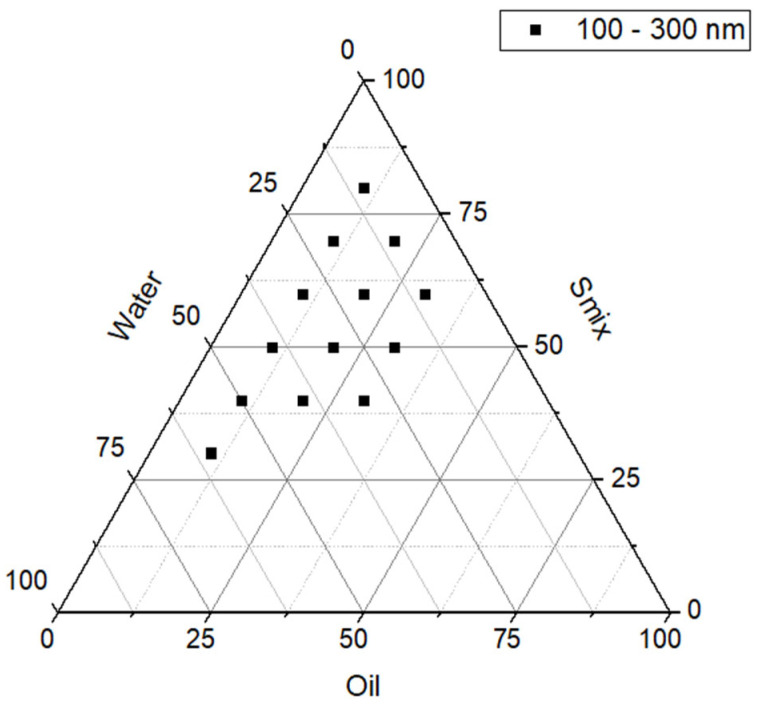
Ternary phase diagram highlighting the 14 stable nanoscale emulsions (100−300 nm) without phase separation after optimization.

After preparing the 59 formulations, 14 emulsions demonstrated stability without phase separation, indicating robust and consistent systems. These formulations were selected for further evaluation by incorporating silibinin (SLB) to assess potential changes in their visual and physical properties. As shown in Figure 4, the 14 selected formulations correspond to the points within the diagram that resulted in stable emulsions in the nanoscale range, with droplet sizes between 100 and 300 nm.

The stable formulations were observed in areas where water content ranged from 10 to 70%, oil content was between 10 and 30%, and Smix content varied from 20 to 80%. These specific ranges highlight the critical role of each component in forming a stable nanoemulsion. Water, as the continuous phase in oil-in-water emulsions, is essential for dispersing the oil droplets uniformly. A moderate-to-high water content provides sufficient medium for droplet dispersion, but excessive water dilutes the Smix concentration, reducing its ability to stabilize the interface. Conversely, low water content can lead to insufficient dispersion and increased viscosity, resulting in phase separation.

The oil phase, composed of Capryol 90, was found to be effective within a range of 10 to 30%. This moderate oil proportion ensures that the Smix system can emulsify the droplets efficiently without overwhelming the stabilizing capacity of the surfactants. Higher oil concentrations tend to increase droplet coalescence, destabilizing the emulsion, while lower oil content limits the dispersed phase, potentially affecting the functionality of the emulsion.

The Smix content in stable formulations ranged from 20% to 80%, where the surfactant and cosurfactant combination effectively, reduced interfacial tension, and formed a cohesive stabilizing layer around the oil droplets. The surfactants, with their hydrophilic heads and hydrophobic tails, align at the oil–water interface, reducing the interfacial energy and stabilizing the dispersed oil phase. The cosurfactant, typically less hydrophilic, enhances the fluidity at the interface, allowing the surfactant molecules to pack more efficiently and prevent droplet coalescence. This interaction is critical for achieving a uniform nanoscale droplet size distribution and long-term stability.

The stable nanoemulsions observed in the diagram demonstrate how the interplay between water, oil, and Smix proportions determines the emulsion’s stability. Excessive water dilutes the system, reducing emulsification efficiency, while excessive oil increases the likelihood of droplet coalescence and phase separation. The identified stable regions indicate a selected balance of these components, leading to the formation of robust emulsions with high stability and nanoscale droplet sizes.

### 3.4. Stability Studies

#### 3.4.1. Accelerated Stability Testing

Accelerated stability testing was conducted on 14 emulsion formulations to evaluate their robustness under extreme conditions, including centrifugation and heating–cooling cycles. These tests were designed to simulate accelerated aging and identify formulations capable of maintaining their structural and physicochemical integrity over time. The visual appearance, average particle size, and PDI of the emulsions were monitored throughout the tests.

Upon completing the evaluations, only 2 of the 14 formulations demonstrated structural and physicochemical stability, showing no significant changes in particle size, PDI, or visual appearance. These results indicate that the two formulations possess the necessary robustness to withstand challenging conditions while maintaining their colloidal structure. The compositions of the stable emulsions are detailed in Table 2, which also highlights their proportions of oil, Smix, and water, along with the average particle diameters and PDI values after the accelerated stability testing.

**Figure 5:**
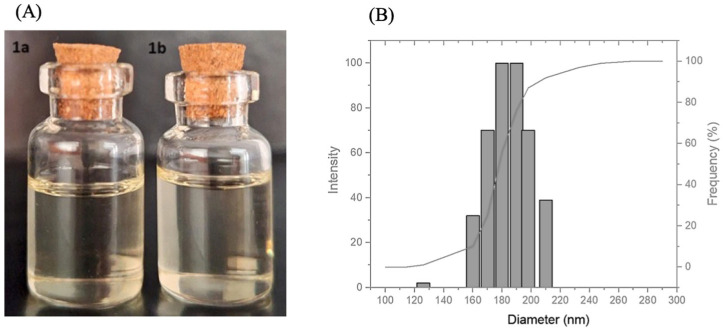
(**A**) Visual appearance of nanoemulsions: (**1a**)—nanoemulsion containing SLB, (**1b**)—nanoemulsion without SLB. (**B**) Particle size distribution of the nanoemulsion containing SLB, showing a predominant diameter range of 160–220 nm.

For further optimization, Formulation 1 was selected due to its favorable composition, which features a higher water fraction and reduced Smix ratio compared to Formulation 2, despite both formulations exhibiting similar average particle sizes, PDI, zeta potential (ZP), and encapsulation efficiency (EE%). The final formulation displayed a transparent appearance and no phase separation (Figure 5), even after being subjected to the rigorous accelerated stability tests, meeting the standards for a high-quality emulsion.

The oil-in-water emulsion composed of Capryol, Tween 80, and Transcutol achieved an encapsulation efficiency of 98% for SLB, representing a near-complete encapsulation that surpasses typical values for similar nanoemulsion systems by approximately 20%. This high encapsulation rate underscores the effectiveness of these components in forming a stable emulsion. Capryol, in combination with Tween 80 and Transcutol, plays a pivotal role in facilitating the transport of the lipophilic SLB within the emulsion’s internal phase. These components not only protect the SLB from premature degradation but also may enhance its bioavailability, making the emulsion a promising platform for drug delivery [30,31].

Interestingly, the stability of the emulsions was observed to persist even with a zeta potential near neutrality. While conventional wisdom suggests that a non-zero zeta potential indicates greater stability, the use of nonionic surfactants and cosurfactants in these formulations likely contributed to their robustness. Nonionic surfactants, such as Tween 80, are known for their ability to form a stable adsorbed layer at the droplet interface. This layer reduces interfacial tension and creates a physical barrier that prevents droplet coalescence, thereby maintaining colloidal stability over time [32].

Although nonionic surfactants do not significantly alter the zeta potential, their stabilizing effects arise from the physical adsorption layer rather than electrostatic repulsion. As a result, the emulsions exhibit stability despite their ZP values being close to neutrality, confirming their suitability for pharmaceutical and industrial applications [33].

Figure 5 provides a detailed analysis of the particle size distribution of Formulation 1. Most particles are concentrated within an average diameter range of 160 to 220 nm, with a peak intensity between 180 and 200 nm. This sharp distribution suggests high uniformity in the production process, minimizing variability and enhancing the consistency of the final product. The cumulative frequency curve further highlights the predominant size range, with most particles falling between 160 and 200 nm. The absence of significant fractions of very small or very large particles confirms the efficiency of the production process, which contributes to the overall stability and performance of the emulsion.

In summary, the accelerated stability testing demonstrated that Formulation 1 is a robust and reliable candidate for further development. Its high encapsulation efficiency, stability under extreme conditions, and consistent particle size distribution highlight its potential as a high-quality platform for delivering lipophilic bioactive compounds like SLB.

#### 3.4.2. Storage Stability

**Figure 6:**
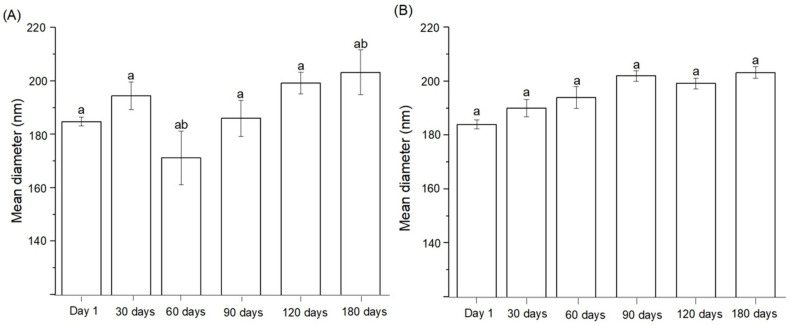
Stability of SLB-loaded nanoemulsions: average particle size measured over 180 days at room temperature (**A**) and refrigerated conditions (**B**). Results demonstrate the stability of the nanoemulsion under both storage conditions. ^a,b,^ Same letters mean statistical equality and different letters statistical inequality (ANOVA and post-Tukey test and *p* < 0.05).

The stability of the silibinin-loaded nanoemulsion was evaluated over 180 days under two storage conditions: room temperature (Figure 6A) and refrigeration (Figure 6B). The average particle diameter was measured at regular intervals to assess potential changes in the structural integrity of the nanoemulsion.

At room temperature (Figure 6A), the nanoemulsion exhibited minimal variations in particle size over the storage period. The average particle diameter remained within the nanoscale range (approximately 200 nm), and no significant differences were observed in most intervals, as indicated by the overlapping statistical groupings (denoted by “a”). A slight reduction in particle size was noted at 60 days, followed by a return to initial values at 180 days, reflecting the dynamic equilibrium of the emulsion system under ambient conditions. These results indicate that the nanoemulsion was able to maintain its structural stability and avoid destabilizing phenomena such as coalescence or Ostwald ripening over the six-month period [34].

In refrigerated conditions (Figure 6B), the nanoemulsion demonstrated exceptional stability, with particle size variations remaining within ±5% over a storage period of 180 days, ensuring a shelf-life improvement of up to 2-fold compared to emulsions stored at room temperature. The average particle diameter remained consistent at approximately 200 nm, and the absence of variability suggests that lower temperatures slowed any destabilization processes. Refrigeration appears to enhance the stability of the nanoemulsion by reducing molecular motion and the likelihood of droplet interaction, further preventing coalescence.

The comparison between the two storage conditions highlights the robustness of the nanoemulsion. While room temperature storage showed slight variations in particle size at certain time points, these changes were minor and did not impact the overall integrity of the emulsion. Refrigerated storage, however, provided an additional layer of stability, maintaining uniform particle size distribution throughout the 180-day period.

These findings emphasize the potential of the silibinin-loaded nanoemulsion as a stable drug delivery platform, suitable for varying storage conditions. The consistency in particle size under both room temperature and refrigerated conditions underscores the formulation’s robustness and long-term reliability, making it a promising candidate for pharmaceutical applications.

### 3.5. Quantitative Mucosal Adhesion Analysis by QCM-D

**Figure 7:**
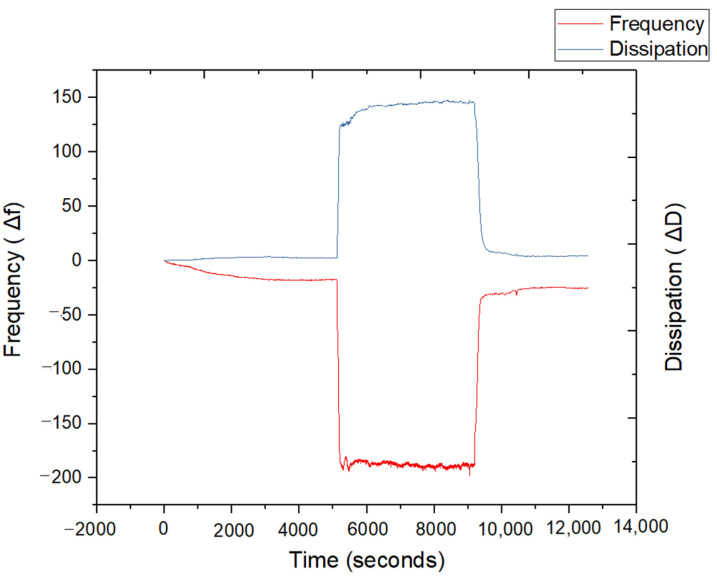
QCM-D analysis of nanoemulsion–mucin interactions: frequency (Δf) and dissipation (AD) shifts over time.

The QCM-D analysis was performed to evaluate the interaction between the nanoemulsion and mucin, focusing on frequency (Δf) and dissipation (ΔD) changes over time [35]. Figure 7 provides insights into the adsorption, structural rearrangement, and detachment of the nanoemulsion on the mucin layer.

A significant decrease in frequency is observed upon the introduction of the nanoemulsion (around 2000 s), indicating mass adsorption onto the mucin-coated surface. This suggests a strong interaction between the nanoemulsion and the mucin layer, consistent with the mucoadhesive nature of the formulation. The extent of frequency reduction reflects the extent of mass deposition, including both the nanoemulsion droplets and any associated hydration layer. Concurrently, there is a notable increase in dissipation, indicative of a soft and viscoelastic film forming on the mucin layer due to the adsorption of the nanoemulsion. This behavior supports the hypothesis that the nanoemulsion interacts dynamically with the mucin, forming a hydrated layer that enhances retention and mucoadhesion. After the initial adsorption phase, both frequency and dissipation stabilize, indicating that equilibrium has been reached. This suggests that the nanoemulsion forms a stable interaction with the mucin membrane without further significant rearrangements. Finally, when the nanoemulsion is rinsed (around 9000 s), the frequency increases and dissipation decrease sharply, indicating mass desorption. However, the magnitude of the frequency recovery is less than the initial baseline, suggesting that a fraction of the nanoemulsion remains bound to the mucin layer, demonstrating strong mucoadhesion.

The results confirm the mucoadhesive properties of the nanoemulsion, likely driven by hydrophobic interactions and hydrogen bonding between the formulation components and the mucin layer. The increase in dissipation highlights the viscoelastic nature of the nanoemulsion–mucin interaction, which is advantageous for prolonged retention in mucosal environments. These findings align with the intended application of the nanoemulsion as a drug delivery system, providing enhanced bioavailability through sustained interaction with mucosal surfaces [35,36].

### 3.6. Rheological Properties Study

**Figure 8:**
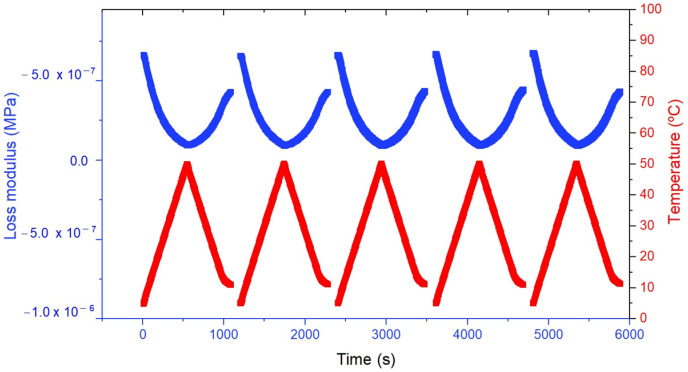
Temperature ramp of the selected nanoemulsion. The graph depicts the variation in the loss modulus (MPa) and temperature (°C) as a function of time (s). The loss modulus is represented by the blue line, while the temperature is represented by the red line.

The temperature sweep analysis (Figure 8) highlights the thermal response of the nanoemulsion by monitoring changes in the loss modulus (G″) across multiple thermal cycles. The results demonstrate a strong dependence of the material’s viscoelastic properties on temperature variations. As the temperature increases from 25 °C to 90 °C, the loss modulus decreases, reflecting a reduction in the viscous component of the nanoemulsion. This behavior indicates that the material becomes more fluid-like at elevated temperatures, which is typical for formulations that soften or exhibit increased energy dissipation when heated. Upon cooling, the loss modulus consistently returns to its original state, confirming the reversible thermal response and the nanoemulsion’s ability to maintain structural integrity through multiple heating and cooling cycles.

The stability of the loss modulus oscillations over repeated thermal cycles emphasizes the thermal robustness of the nanoemulsion. The absence of significant degradation in viscoelastic properties underscores its ability to withstand thermal stress without compromising its functional characteristics. This stability is crucial for applications requiring consistent performance under varying temperatures, ensuring that the material remains reliable and durable over time [14].

The observed retention of viscoelastic properties despite temperature fluctuations further validates the nanoemulsion’s potential for mucoadhesive applications. In mucosal environments, where physiological temperature variations are common, maintaining viscoelastic behavior is essential for prolonged retention and effective drug delivery. These findings underscore the importance of thermal analysis in evaluating the performance of nanoemulsions, particularly in scenarios where stability and functionality under dynamic conditions are critical. The SLB-loaded nanoemulsion demonstrates a high level of adaptability and robustness, making it a promising candidate for advanced drug delivery systems targeting mucosal surfaces. 

**Figure 9:**
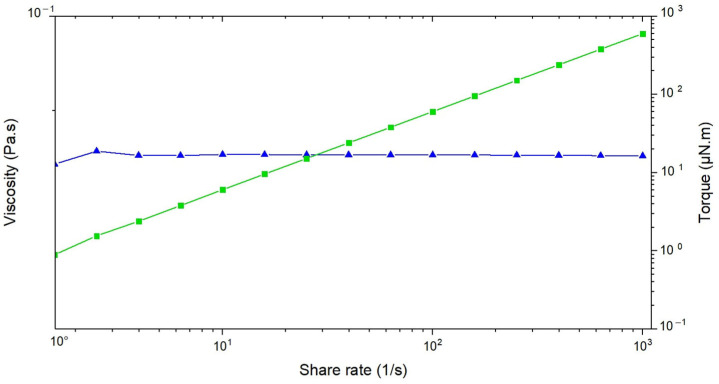
Graph showing the relationship between shear rate, viscosity, and torque. Viscosity (blue triangles) increases linearly with shear rate, while torque (green squares) remains constant. Both y-axes are on a logarithmic scale.

The flow sweep analysis presented in Figure 9 evaluates the rheological behavior of the nanoemulsion under increasing shear rates, with viscosity represented in blue and torque in green. The data demonstrate that the viscosity remains constant across all shear rates, confirming that the nanoemulsion behaves as a Newtonian fluid. This constancy suggests a uniform internal structure, where the components maintain their arrangement and resist significant rearrangement under applied stress. The absence of shear-thinning or thickening phenomena indicates that the nanoemulsion’s viscosity is independent of shear rate, a property that contributes to its predictable flow behavior [37].

The torque increases linearly with shear rate, which is characteristic of Newtonian fluids. This proportional relationship further confirms the mechanical stability of the nanoemulsion. The steady increase in torque without deviation reflects the robustness of the formulation and its ability to maintain consistent properties even under mechanical stress.

From a drug delivery perspective, the Newtonian behavior of the nanoemulsion ensures predictable and reproducible flow properties, making it straightforward to process and apply. While it does not exhibit shear-thinning behavior, the constant viscosity allows for uniform spreading over mucosal surfaces. This consistency supports the formulation’s ability to provide stable and reliable drug delivery, ensuring even distribution and prolonged retention on mucosal tissues.

Overall, the nanoemulsion’s Newtonian fluid behavior, characterized by constant viscosity and a linear torque response, underscores its stability and adaptability for mucosal drug delivery applications. The rheological properties ensure ease of handling and efficient application, further enhancing the formulation’s potential as a robust platform for therapeutic delivery.

### 3.7. Determination of Surface Tension

**Figure 10:**
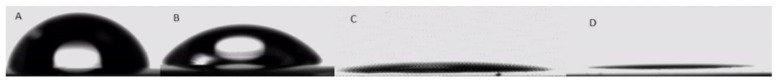
Drops illustrating different contact surfaces: (**A**) gold, (**B**) after mucin adsorption, (**C**) white sample and its interaction with mucin, and (**D**) SLB-loaded sample and its interaction with mucin.

The contact angle analysis, as shown in Figure 10 and Table 3, provides critical insights into the wettability and interaction of various surfaces with water droplets, highlighting the surface tension properties. The contact angle measured on a gold crystal (Surface A) was 99.0°, indicating low wettability and a strongly hydrophobic surface. This behavior is attributed to the inert and non-polar nature of gold, which weakly interacts with water molecules, resulting in a large droplet curvature.

After mucin adsorption onto the gold surface (Surface B), the contact angle decreased significantly to 53.7°, reflecting moderate wettability. This change is due to the amphiphilic nature of mucin, which contains both hydrophilic and hydrophobic regions. The presence of hydrophilic glycoproteins in mucin promotes stronger interactions with water molecules, thereby reducing the contact angle and confirming successful mucin adsorption.

For the white nanoemulsion interacting with mucin (Surface C), the contact angle decreased further to 10.3°, indicating extremely high wettability. This suggests that the white nanoemulsion forms a strong hydrophilic network upon contact with mucin, enhancing wettability and reducing surface tension. Finally, the SLB-loaded nanoemulsion (Surface D) exhibited the smallest contact angle of 6.2°, indicating almost complete spreading of the water droplet. This substantial reduction in the contact angle highlights the enhanced interaction between the SLB-loaded nanoemulsion and mucin, likely driven by the amphiphilic components of the nanoemulsion that interact with the hydrophilic regions of mucin.

These findings emphasize the progressive reduction in contact angle from Surface A to Surface D, reflecting the increasing hydrophilic nature of the surfaces. The SLB-loaded nanoemulsion demonstrated the highest wettability, suggesting strong mucoadhesive potential. The interaction of surfactants in the nanoemulsion with mucin likely plays a critical role in drastically reducing surface tension, facilitating enhanced adhesion to mucosal tissues.

The contact angle analysis highlights the remarkable mucoadhesive properties of the SLB-loaded nanoemulsion. The significant reduction in contact angle, coupled with enhanced wettability, demonstrates strong interactions with mucin layers. These results underscore the potential of SLB-loaded nanoemulsions as robust platforms for mucosal drug delivery, ensuring effective adhesion and prolonged retention on biological surfaces [38,39].

## 4. Conclusions

This study successfully developed and characterized silibinin (SLB)-loaded nanoemulsions as a promising platform for mucosal drug delivery. The selected formulations, prepared using the spontaneous emulsification method, demonstrated excellent physicochemical properties, including nanoscale particle sizes (100–300 nm), a low PDI, a high encapsulation efficiency, and stability under varying conditions. The combination of Capryol 90, Tween 80, and Transcutol in carefully selected proportions proved effective in overcoming the solubility and bioavailability challenges associated with SLB. The use of pseudoternary phase diagrams enabled the precise identification of compositional ranges that yielded stable nanoemulsions with selected droplet size and uniformity. Rheological analysis confirmed Newtonian behavior, ensuring the formulations’ mechanical stability and ease of application. Moreover, the evaluation of mucoadhesive properties using quartz crystal microbalance with dissipation (QCM-D) revealed strong interactions with mucin, indicating enhanced retention at mucosal sites and potential for prolonged therapeutic effects. The reduction in surface tension and improved wettability further validated the nanoemulsion’s suitability for mucosal application. These findings highlight the robustness and versatility of SLB-loaded nanoemulsions as an effective mucosal drug delivery system.

## Figures and Tables

**Table 1:** HLB, appearance, particle size, polydispersity index (PDI), and zeta potential of tested formulations with different Smix ratios (Tween 80–Transcutol).

Smix Ratio	HLB	Appearance	Size (nm)/PDI	Zeta Potential (mV)
1:1	9.6	translucent emulsion without phase separation	343/0.351	−9.66
1:2	7.87	translucent emulsion with phase separation	592/0.270	−11.3
1:3	6.9	translucent emulsion with phase separation	942/0.383	−12.0
1:4	6.36	translucent emulsion with phase separation	563/0.369	−15.2
1:5	6.04	white emulsion with phase separation	256/0.216	−15.2
1:6	5.71	white emulsion with phase separation	622/0.379	−12.6
1:7	5.60	white emulsion with phase separation	318/0.344	−15.9
1:8	5.39	white emulsion with phase separation	212/0.250	−15.1
1:9	5.28	white emulsion with phase separation	486/0.330	−15.9
2:1	11.32	white emulsion with phase separation	968/0.438	−14.9
3:1	12.3	white emulsion with phase separation	419/0.351	−10.9
4:1	12.84	white emulsion with phase separation	481/0.394	−10.2
5:1	13.16	white emulsion with phase separation	492/0.319	−11.2
6:1	13.49	white emulsion with phase separation	296/0.364	−9.27
7:1	13.6	white emulsion with phase separation	806/0.364	−9.31
8:1	13.8	white emulsion with phase separation	1060/0.375	−12.9
9:1	13.92	white emulsion with phase separation	794/0.423	−10.4

**Table 2:** Composition of stable formulations and physical characteristics after accelerated stability testing.

Formulation	Oil (%)	Smix (%)	Water (%)	Size (nm)	PDI	ZP (mV)	EE (%)
1	20	60	20	204 (20)	0.276 (0.008)	−11.5	98
2	20	70	10	203 (14)	0.274 (0.043)	−10.5	98

**Table 3:** Contact angles of various samples.

Sample	Contact Angle
A—Gold crystal	99.0°
B—Crystal with mucin	53.7°
C—White emulsion	10.3°
D—SLB-loaded emulsion	6.2°

## Data Availability

Data will be available on request.
